# The impact of school-based screening on service use in adolescents at risk for mental health problems and risk-behaviour

**DOI:** 10.1007/s00787-022-01990-z

**Published:** 2022-04-30

**Authors:** Sophia Lustig, Michael Kaess, Nina Schnyder, Chantal Michel, Romuald Brunner, Alexandra Tubiana, Jean-Pierre Kahn, Marco Sarchiapone, Christina W. Hoven, Shira Barzilay, Alan Apter, Judit Balazs, Julio Bobes, Pilar Alejandra Saiz, Doina Cozman, Padraig Cotter, Agnes Kereszteny, Tina Podlogar, Vita Postuvan, Airi Värnik, Franz Resch, Vladimir Carli, Danuta Wasserman

**Affiliations:** 1 Institute of Psychology, University of Heidelberg, Heidelberg, Germany; 2 Department of Child and Adolescent Psychiatry, University Hospital Heidelberg, Heidelberg, Germany; 3 University Hospital of Child and Adolescent Psychiatry and Psychotherapy, University of Bern, Bern, Switzerland; 4 School of Public Health, The University of Queensland, Brisbane, Australia; 5 Policy and Epidemiology Group, Queensland Centre for Mental Health Research, Brisbane, Australia; 6 Clinic of Child and Adolescents Psychiatry, Psychosomatics and Psychotherapy, University of Regensburg, Regensburg, Germany; 7 Department of Psychiatry and Clinical Psychology, Centre Psychothérapique de Nancy, Nancy, France; 8 Université de Lorraine, Nancy, France; 9 Department of Medicine and Health Science, University of Molise, Campobasso, Italy; 10 National Institute for Health, Migration and Poverty, Rome, Italy; 11 Division of Child and Adolescent Psychiatry, New York State Psychiatric Institute, Columbia University, New York, NY USA; 12 Feinberg Child Study Centre, Schneider Children’s Medical Centre, Tel Aviv University, Tel Aviv, Israel; 13 Department of Community Health, University of Haifa, Haifa, Israel; 14 Institute of Psychology, Eötvös Loránd University, Budapest, Hungary; 15 Bjørknes University College, Oslo, Norway; 16 Department of Psychiatry, Centro de Investigación Biomédica en Red de Salud Mental, University of Oviedo, Oviedo, Spain; 17 Clinical Psychology Department, Iuliu Hatieganu University of Medicine and Pharmacy, Cluj-Napoca, Romania; 18 Child and Adolescent Mental Health Services North Cork Area, HSE South, Mallow, Ireland; 19 Slovene Center for Suicide Research, Andrej Marusic Institute, University of Primorska, Koper, Slovenia; 20 Estonian-Swedish Mental Health and Suicidology Institute, Tallinn, Estonia; 21 Tallinn University School of Natural Science and Health, Tallinn, Estonia; 22 Department of Public Health Sciences, Methods Development and Training in Suicide Prevention, National Swedish Prevention of Mental Ill-Health and Suicide (NASP)WHO Collaborating Centre for ResearchKarolinska Institute, Stockholm, Sweden

**Keywords:** Adolescents, Mental health problems, Risk behaviours, School-based screening, Service use

## Abstract

Early detection and intervention can counteract mental disorders and risk behaviours among adolescents. However, help-seeking rates are low. School-based screenings are a promising tool to detect adolescents at risk for mental problems and to improve help-seeking behaviour. We assessed associations between the intervention “Screening by Professionals” (ProfScreen) and the use of mental health services and at-risk state at 12 month follow-up compared to a control group. School students (aged 15 ± 0.9 years) from 11 European countries participating in the “Saving and Empowering Young Lives in Europe” (SEYLE) study completed a self-report questionnaire on mental health problems and risk behaviours. ProfScreen students considered “at-risk” for mental illness or risk behaviour based on the screening were invited for a clinical interview with a mental health professional and, if necessary, referred for subsequent treatment. At follow-up, students completed another self-report, additionally reporting on service use. Of the total sample (*N* = 4,172), 61.9% were considered at-risk. 40.7% of the ProfScreen at-risk participants invited for the clinical interview attended the interview, and 10.1% of subsequently referred ProfScreen participants engaged in professional treatment. There were no differences between the ProfScreen and control group regarding follow-up service use and at-risk state. Attending the ProfScreen interview was positively associated with follow-up service use (OR = 1.783, 95% CI = 1.038–3.064), but had no effect on follow-up at-risk state. Service use rates of professional care as well as of the ProfScreen intervention itself were low. Future school-based interventions targeting help-seeking need to address barriers to intervention adherence.

Clinical Trials Registration: The trial is registered at the US National Institute of Health (NIH) clinical trial registry (NCT00906620, registered on 21 May, 2009), and the German Clinical Trials Register (DRKS00000214, registered on 27 October, 2009).

**Supplementary Information:**

The online version contains supplementary material available at 10.1007/s00787-022-01990-z.

## Introduction

Mental disorders cause a high burden in children and adolescents. Among the ten leading causes of disease burden in 10–24 year-olds, five are related to mental and substance use disorders [1]. Another four, such as road traffic accidents and HIV/AIDS [1], may be directly or indirectly related to risk behaviour. Furthermore, risk behaviours and poor mental health of young people are often correlated [2–6]. For example, adolescents’ depressive symptoms are associated with multiple risk behaviours [7]. Early detection and intervention might reduce the burden of mental disorders for individuals and societies [8]. Since many lifetime mental disorders begin in childhood or adolescence [9, 10] and often continue through the life course, early detection and subsequent intervention has an even bigger impact in this age group [8].

Despite the high need, young peoples’ help-seeking behaviour within the mental healthcare system is remarkably low [11–14]. These low help-seeking rates might be one reason why the burden of mental disorders does not reduce in children and adolescents. The focus on how young people’s help-seeking behaviour could be increased is thus warranted. School-based screenings may be promising tools to detect young people at-risk for mental health problems and risk behaviour [15–19] that are sometimes not otherwise identified [20]. Accordingly, they have the potential to increase subsequent help-seeking behaviour [11], and thus indirectly reduce mental health problems. Schools are an obvious and acceptable environment for prevention and intervention [17, 21] and school-based mental health professionals are perceived helpful by high-school students [22]. School-based screenings usually involve two stages [17, 23, 24] and have shown to be clinically valid and reliable [25, 26]. First, all students complete a brief self-report screening instrument to detect those at-risk for mental problems or risk behaviour. Second, those considered at-risk, based on the self-report, are invited to attend a clinical face-to-face interview with a mental health professional; this aims to identify those that require ongoing support [17, 27] and, if needed, refers them to a subsequent intervention.

School-based screenings addressing current suicidality have shown to be associated with help-seeking at a later time [11]. If screenings addressing a wider array of mental health problems and risk behaviours are associated with help-seeking in a similar way is yet unknown. School-based screenings are a crucial part of indicated preventions aiming at individuals with subclinical symptoms. They might not only be associated with professional service use but also, at least indirectly, with follow-up at-risk states. To the best of our knowledge, this has not yet been studied.

Within the framework of the “Saving and Empowering Young Lives in Europe” (SEYLE) study [28], a two-stage school-based screening for mental health problems and risk behaviour was implemented in a large sample of European adolescents. The present study reports on the one-year follow-up of participants that were randomly assigned to either the two-staged screening intervention “Screening by Professionals” (ProfScreen) or the control group. For the present study, only those participants who were classified as being ‘at-risk’ for mental illness or risk behaviour in the baseline screening were examined, as for these participants, seeking professional help would be appropriate and necessary.

We aimed to illustrate advantages and disadvantages of school-based screenings by addressing following research questions:

- (1) Compared with the control group, is allocation to the ProfScreen intervention associated with higher levels of professional service use at follow-up?
- (2) Compared with the control group, is allocation to the ProfScreen intervention associated with reduced risk for mental illness or risk behaviour at follow-up (i.e. reduced frequency of at-risk state)?

## Methods

### Study design

**Fig.1:**
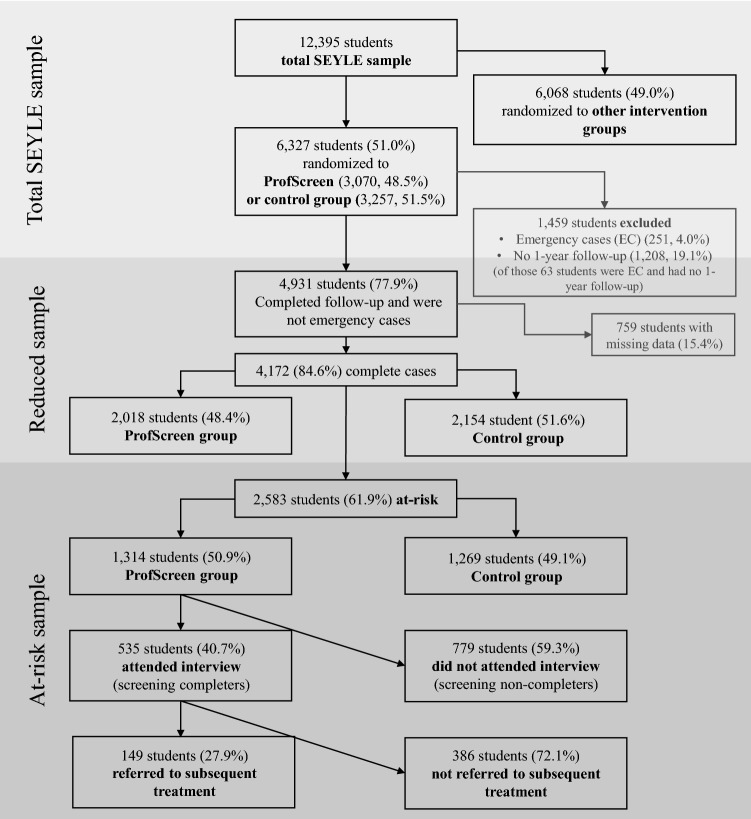
Flow-chart of recruitment and participation of students in the SEYLE study, participation on screening process at baseline (11/2009–12/2010) and completion of follow-up questionnaire (12 month after baseline)

The SEYLE study is aimed at the prevention and early intervention of mental problems, suicide, and risk behaviours [registered at the US National Institute of Health (NIH) clinical trial registry (NCT00906620), and the German Clinical Trials Register (DRKS00000214)]. SEYLE is a randomized controlled trial (RCT) including three different school-based interventions and one control group. For the present study, only participants who were randomized to either the ProfScreen intervention or the control group were included to examine the effectiveness and feasibility of this particular two-staged intervention, which had specifically been developed to facilitate referral to appropriate professional mental healthcare. Wasserman and colleagues described details of methodology and interventions of the SEYLE study, including the other two intervention groups, the gatekeeper training “Question, Persuade, and Refer” (QRF) and the awareness training “Youth Aware of Mental Health Programme” (YAM) [28]*.* Eleven countries including Austria, Estonia, Germany, France, Hungary, Ireland, Israel, Italy, Romania, Slovenia, and Spain implemented the SEYLE study, with Sweden as the coordinating centre. Ethical approval was granted locally to each study site. The selection of the countries allowed for a broad geographical representation of Europe. In each country, researchers randomly selected mixed-gender post-primary schools within a pre-determined and representative study site. Of the total 264 schools that were approached for participation, 179 schools accepted (overall response rate was 67.8%). The participating schools were randomly assigned to one of the three interventions or to the control group. Only one type of intervention was performed in each school to avoid contamination and confounding. Students and teachers were only aware of the respective intervention arm implemented at their school, without being informed of other intervention arms implemented at other schools. Assessments and interventions were homogenous and robust across countries (for more details on methods including randomisation process of the SEYLE study, see [29]). Inclusion criteria for the current study were: (1) being randomised to either ProfScreen or control group, and (2) screening positive for mental health problems and/or risk behaviour at baseline (in the following labelled as being ‘at-risk’). Students that reported current suicidality at baseline (emergency cases) received an immediate, special intervention [25]. They remained in the study but were excluded from analyses (Fig. 1).

### Screening by Professionals (ProfScreen) and control group/minimal intervention

The ProfScreen intervention was designed to identify students at-risk for mental problems or risk behaviours and followed a two-stage screening process: (1) students’ self-report; (2) clinical evaluation and referral to a healthcare service for treatment, if necessary. The University of Heidelberg and the National Swedish Prevention of Mental Ill-Health and Suicide (NASP) at the Karolinska Institutet developed this intervention, and it was pilot tested in Heidelberg. Students of the ProfScreen group that screened at or above at least one of the eleven pre-defined cut-off points in the school-based screening (e.g. BMI < 16.5, > 4 incidents of bullying in the last year, > 5 h media exposure per day; for all cut-offs see Online Resource 1; all Online Resources are provided in online Supporting Information) [23, 28] were considered being at-risk for mental illness or risk behaviour. These participants were invited to attend a clinical semi-structured interview with a psychologist or psychiatrist. The interview was developed based on the Schedule for Affective Disorders and Schizophrenia for School-Age Children (K-SADS) [30]. It was designed to distinguish between students that required further mental healthcare due to their psychological problems and those who did not, rather than determining clinical diagnoses. For ethical reasons, the control group received minimal intervention comprising six educational posters displayed in the class rooms [25]. Both ProfScreen and minimal intervention took place within four weeks after the baseline assessment.

### School-based assessments

For the first stage of the screening process, students completed a 60–90 min self-report questionnaire in a school-based setting, on mental health problems (depression, anxiety, suicidal tendencies, non-suicidal self-injury, and eating behaviour) and risk behaviours (sensation seeking, delinquent behaviour, substance abuse, media exposure, social relationships, bullying, and school attendance). This baseline questionnaire additionally assessed students’ socio-demographics [28]. The instruments used were validated and/or used in previous studies (see Online Resource 1). Several child and adolescent psychologists and psychiatrists of the SEYLE consortium agreed on the cut-off points, during a consensus conference.

The same scales were used to assess mental health problems and risk behaviours at 12 month follow-up. Additionally, participants indicated if and what type of service or support they received since the implementation of the ProfScreen or minimal intervention (control group) at baseline. Possible answers included help from health professionals (medication, professional one-on-one therapy, group therapy, or advice from a health professional), and help from the lay support system (healthy lifestyle group or a mentor to talk to).

### Statistical analyses

A flow chart was created with all participants in the SEYLE study to show what percentage were in the ProfScreen or control group, and how many participants fully completed the follow-up questionnaire. Additionally, the number of interview attendees (ProfScreen completers) and subsequent referrals (referred ProfScreen completers) was calculated for the ProfScreen group. In the next step, the sample was reduced to those participants who were identified as being at-risk for mental illness or risk behaviours at baseline screening (see Online Resource 1), as the research questions of the present study (follow-up service use and changes in at-risk status) can only be meaningfully addressed on the basis of participants with existing psychological distress, and only these participants were invited to the stage-two interview. All subsequent data analyses refer to this at-risk subsample. Descriptive statistics were calculated separately for participants of the ProfScreen and the control group regarding socio-demographic variables, screening parameters, and follow-up at-risk state. For baseline differences and effect sizes regarding socio-demographic variables and screening parameters between participants from ProfScreen and the control group, independent t-tests were implemented for continuous variables after confirming that they met the required assumptions. Categorical variables were compared with Chi-square tests.

To evaluate the effects of the ProfScreen intervention on at-risk state and professional service use, the binary variable ‘at-risk follow-up’ was created, describing whether participants did (yes), or did not meet (no) at least one of the eleven pre-defined cut-off points at follow-up (see Online Resource 1). The variable ‘service use’ reflected if the participants received help from a health professional (yes), or if they sought help within the lay support system or did not seek any help (no). Since this study focuses on the use of professional help, ‘service use’ in the following always refers to the use of specialist mental health services (psychologists, psychiatrists, psychiatric clinics). Simultaneous logistic regression was used to model the effect of the ProfScreen intervention on follow-up service use (research question 1; adjusted for age, sex, and baseline screening parameters) and on follow-up at-risk state (research question 2; adjusted for age and sex only, as baseline screening parameters were part of the criteria for at-risk status).

In post-hoc analyses, the variable ‘ProfScreen completer’ was defined to distinguish within the ProfScreen group between participants who completed the intervention per protocol (participated in the stage 2 interview) and those who did not. For baseline differences regarding socio-demographic variables and screening parameters between these groups, independent t-tests were implemented for continuous variables after confirming that they met the required assumptions. Categorical variables were compared with Chi-square tests. In further post-hoc analyses, we examined whether service use and at-risk state differed at follow-up when comparing the control group with the ‘ProfScreen completer’ subgroup, to find out if the ProfScreen intervention had an effect compared to the control group when the intervention was performed per protocol. For this purpose, we computed simultaneous logistic regressions adjusted for age, sex, and baseline screening parameters (regarding follow-up service use), or adjusted for age, sex, and service use (regarding follow-up at-risk state) on the new variable ‘ProfScreen per protocol’ (yes = ProfScreen completer, no = control group).

Each variable had between 0 and 8.7% missing values (see Online Resource 2). First, we removed participants with missing age and sex. Second, we analysed patterns of the missing outcome follow-up at-risk state according to age, sex, intervention group, and country. Then, we analysed complete cases. Results with *p* ≤ 0.05 were considered statistically significant. The statistical analyses were performed using Stata version 15 (Stata Corporation, College Station, TX, USA).

## Results

### Description of samples and baseline differences between the ProfScreen and control group

Of the total *N* = 12,395 SEYLE study participants, 3070 were randomised to the ProfScreen and 3257 to the control group. Of those, 4172 (65.9%) completed the 12 month follow-up, were not emergency cases, and had complete data. Among those complete cases, 2583 (61.9%) students were considered at-risk for mental problems or risk behaviour at baseline; comprising 1314 (50.9%) students of the ProfScreen and 1269 (49.1%) of the control group. 535 (40.7%) students of the ProfScreen group attended the clinical interview and 149 (27.9%) of these were referred to subsequent treatment (Fig. 1).

Subsequent data analyses refer to the 2583 students that were at-risk for mental health problems or risk behaviour at baseline. Compared to the control group, students of the ProfScreen group screened more often positive for suicidal tendencies and problems in social relationships at baseline (Table 1). The effect sizes of these differences were small. Sex, age, and all other baseline screening parameters did not differ between the ProfScreen and control group (Table 1).

**Table 1:** Sociodemographic and clinical characteristics of the total at-risk sample and statistical comparison of the ProfScreen and control group

	Total at-risk sample (*N* = 2583)	ProfScreen group (*n* = 1314)	Control group (*n* = 1269)	Statistics^b^ *χ*^2^_(df)_, *p*, Cramer’s *V*^c^ / t_(df)_, *p*, Cohen’s *d*^d^
Sex: n (%) Female	1422 (55.1)	743 (52.3)	679 (47.7)	$\chi_{\left( 1 \right)}^{2}$=2.408, p=0.121, *V*=0.031
Age: mean ± SD	15 ± 0.9	15 ± 0.9	15 ± 0.9	*t*_(2581)_ = − 0.129, *p* = 0.898, *d* = − 0.005
Baseline screening parameters, *n* (%) yes				
Depression	680 (26.3)	348 (51.2)	332 (48.8)	$\chi_{\left( 1 \right)}^{2}$=0.052, p=0.820, *V*=0.005
Anxiety	250(9.7)	134(53.6)	116 (46.4)	$\chi_{\left( 1 \right)}^{2}$=0.733, p=0.392, *V*=0.017
Suicidal tendencies	509(19.7)	279(54.8)^*^	230 (45.2)^*^	$\chi_{\left( 1 \right)}^{2}$=5.388, p=0.020, *V*=0.046
Non-suicidal self-injury	518(19.3)	278(53.7)	240	$\chi_{\left( 1 \right)}^{2}$=2.241, p=0.134, *V*=0.030
Eating behaviour	176(6.8)	81(46.0)	(46.3)	$\chi_{\left( 1 \right)}^{2}$=2.491, p=0.114, *V*=-0.032
Risky behaviour^a^	313 (12.1)	152 (48.6)	161(51.4)	$\chi_{\left( 1 \right)}^{2}$=0.718, p=0.397, *V*=-0.017
Substance abuse	1456 (56.4)	740(50.8)	716(49.2)	$\chi_{\left( 1 \right)}^{2}$=0.020, p=0.889, *V*=-0.003
Exposure to media	452 (17.5)	216 (47.8)	236 (52.2)	$\chi_{\left( 1 \right)}^{2}$=2.420, p=0.120, *V*=-0.031
Social relationships	204(7.9)	126(61.8)^*^	78(38.2)^*^	$\chi_{\left( 1 \right)}^{2}$=10.551, p=0.001, *V*=0.064
Bullying	328 (12.7)	178(54.3)	150(45.7)	$\chi_{\left( 1 \right)}^{2}$=1.785, p=0.181, *V*=0.027
School attendance	119(4.9)	61(51.3)	58(48.7)	$\chi_{\left( 1 \right)}^{2}$=0.005, p=0.942, *V*=0.001
Interview attended, n (%) *yes*	*NA*	535(40.7)	*NA*	*NA*
Referred ProfScreen completers, n (%) *yes*	*NA*	149 (11.3)	*NA*	*NA*

*NA* not applicable: interview attendance and referral to further treatment is only applicable to ProfScreen group, *p* p-value, *χ*^*2*^_*(df)*_ Chi-squared test for categorical data with degrees of freedom, *t*_*(df)*_ independent t-test with degrees of freedom

^*^Residuals in cells > 1.96 or <  − 1.96 (indicates that frequency in cell is significantly larger or smaller than expected)

^a^Sensation seeking and delinquent behaviour

^b^parameter comparisons between ProfScreen and control group

^c^Cramer’s *V* of 0.1, 0.3, and 0.5 represent small, medium, and large effect size, respectively

^d^Cohen’s *d* of 0.2, 0.5, and 0.8 represent small, medium, and large effect sizes; Rosenthal’s *r* of 0.1, 0.2, and 0.5 represent small, medium, and large effect size, respectively

### Effects of the ProfScreen intervention

Of the total 2583 students at-risk for mental health problems or risk behaviour, 93 (3.6%) engaged in professional treatment within one year after the baseline assessment; 53 (4.1%) of the ProfScreen and 40 (3.1%) of the control group. Most of these students engaged in professional one-to-one therapy, followed by medication (see Online Resource 3). Neither follow-up service use (Table 2, unadjusted models in Online Resource 4) nor follow-up at-risk state (Table 3, unadjusted models in Online Resource 5) differed significantly between the ProfScreen and the control group, revealing no overall effects of the ProfScreen intervention.

**Table 2:** Adjusted logistic regression of association between ProfScreen intervention and service use (*n* = 2,583)

	Service use after one year	
	OR	95% CI
ProfScreen group^a^	1.401	0.885–2.218
Age^b^	1.067	0.817–1.394
Sex^c^	1.396	0.835–2.334
Baseline screening parameters^d^		
Depression	**1.958**	**1.137–3.372**
Anxiety	1.215	0.628–2.351
Suicidal tendencies	1.121	0.641–1.962
Non-suicidal self-injury	**2.418**	**1.480–3.972**
Eating behaviour	1.627	0.663–3.996
Risky behaviour^e^	1.483	0.783–2.807
Substance abuse	1.157	0.713–1.878
Exposure to media	1.075	0.577–2.004
Social relationships	0.823	0.386–1.755
Bullying	1.417	0.780–2.525
School attendance	1.177	0.432–3.213

*OR* odds ratio, *CI* confidence interval, statistically significant results are displayed in bold, Pseudo *R*^2^ = 0.064

^a^Reference category: control group

^b^Reference: younger age

^c^Reference category: male

^d^Reference categories: cut-offs for mental problems or risk behaviours not met

^e^Sensation seeking and delinquent behaviour

**Table 3:** Adjusted regression of association between ProfScreen intervention and follow-up at-risk state (*n* = 2,583)

	Follow-up at-risk state	
	OR	95% CI
ProfScreen group^a^	0.988	0.742–1.316
Service use^b^	**2.529**	**1.336–4.787**
Age^c^	1.067	0.963–1.182
Sex^d^	0.722	0.554–0.941

*OR* odds ratio, *CI* confidence interval, statistically significant results are displayed in bold, Pseudo R^2^ = 0.010

^a^Reference category: control group

^b^Reference category: no service use

^c^Reference: younger age

^d^Reference category: male

### Post-hoc investigations for complete ProfScreen participation

Within the ProfScreen intervention group, 40.7% participants took part in the interview offered (stage two of the intervention), referred to as ‘ProfScreen completers’. Post-hoc analyses of possible differences between ProfScreen completers and non-completers revealed that ProfScreen completers were younger (*t*_(2581)_ = 5.22, *p* < 0.001). Looking only at the *n* = 535 ProfScreen completers, 29 (5.4%) engaged in professional treatment. Compared to the control group, ProfScreen completers had higher odds of engaging in service use with a professional, within one year after the intervention (OR = 1.78) (Table 4, unadjusted models in Online Resource 4). Regarding follow-up at-risk state, there were no differences between ProfScreen completers and participants of the control group (Table 5, unadjusted models in Online Resource 5).

**Table 4:** Adjusted logistic regression of association between ProfScreen completion per protocol and service use (*n* = 1,529)

	Service use after one year	
	OR	95% CI
ProfScreen completer^a^	**1.783**	**1.038–3.064**
Age^b^	1.228	0.906–1.663
Sex^c^	1.426	0.784–2.595
Baseline screening parameters^d^		
Depression	**1.914**	**1.032–3.551**
Anxiety	1.293	0.609–2.595
Suicidal tendencies	1.115	0.586–2.122
Non-suicidal self-injury	**2.253**	**1.285–3.551**
Eating behaviour	0.38	0.050–2.825
Risky behaviour^e^	1.224	0.570–2.628
Substance abuse	1.210	0.689–2.125
Exposure to media	1.283	0.641–2.570
Social relationships	0.880	0.384–2.015
Bullying	1.521	0.798–2.898
School attendance	1.142	0.798–2.898

*OR* odds ratio, *CI* confidence interval, statistically significant results are displayed in bold, Pseudo *R*^2^ = 0.077

^a^Reference category: control group participants

^b^Reference: younger age

^c^Reference category: male

^d^Reference categories: cut-offs for mental problems or risk behaviours not met

^e^Sensation seeking and delinquent behaviour

**Table 5:** Adjusted regression of association between ProfScreen completion and follow-up at-risk state (*n* = 1,804)

	Follow-up at-risk state	
	OR	95% CI
ProfScreen completers^a^	0.969	0.681–1.447
Service use^b^	**2.357**	**1.115–4.982**
Age^c^	1.101	0.974–1.245
Sex^d^	**0.723**	**0.555–0.943**

*OR* odds ratio, *CI* confidence interval, statistically significant results are displayed in bold, Pseudo R^2^ = 0.011

^a^Reference category: control group

^b^Reference category: no service use

^c^Reference: younger age

^d^Reference category: male

## Discussion

Our study on the school-based ProfScreen intervention had two main findings: (1) assignment to the ProfScreen intervention per se had no effect on follow-up service use nor at-risk state; (2) participation rates within the ProfScreen intervention were low. In post-hoc analyses, we found that participants who completed the ProfScreen intervention per protocol (ProfScreen completers) were more likely to engage in services with a health professional than participants from the control group. However, service use rates for professional mental health services remained low, even among ProfScreen completers, with only 5.4% engaging in professional treatment. Overall, this study shows how difficult it is to effectively support mentally distressed youth through school-based screenings, and demonstrates potential difficulties that two-stage school-based screenings with clinical evaluation by a professional might face. Although the post-hoc analyses revealed that the ProfScreen intervention itself has the potential to improve professional service use, the structure of the intervention appears to make it difficult for students in need to actually use it.

Assignment to the ProfScreen group per se could not promote help-seeking behavior nor improve follow-up at-risk status compared to the control group. Presumably, this is because fewer than half of the ProfScreen participants completed the intervention per protocol (participating in the stage-two interview, ‘ProfScreen completer’). Assignment to the ProfScreen group without participation in the interview did not yet include a specialized intervention but only the baseline screening, so that no effect on service use or at-risk status was to be expected here. Thus, we need to ask ourselves what has made the ProfScreen interview so difficult to access and how a school-based intervention needs to be structured to enable students to benefit from it. Earlier SEYLE findings showed that more students attended the clinical interview if the waiting times were short and if the interview took place at their school, as opposed to other locations [23]. Interventions that take place in schools, such as school counselling, might additionally increase service use rates of young people. Future studies must take this into account when planning screening interventions by improving the second stage of the screening to increase interview attendance and subsequent service use rate. Post-hoc comparisons of the ProfScreen completers and non-completers in the present study also showed that participants were more likely to participate in the interview if they were younger. It is possible that the ProfScreen intervention was more appealing in its design to younger students, and that older students need a different form of support.

The problem that help for mental health problems is not offered in a way that adolescents can readily accept does not only affect ProfScreen intervention, but professional help services in general. Overall service use rates for adolescents at-risk for mental health problems in the present study were low with only 3.6% seeking professional help. Looking only at participants who were referred to subsequent treatment after the ProfScreen interview, and were thus verifiably in high need of professional treatment, the proportion of adolescents who had received appropriate care after one year was 10%. These low help-seeking rates are alarming, yet not unexpected. Previous research has repeatedly pointed to the significant gap between adolescents in need and those receiving professional care [11, 23, 31]. Possible barriers keeping adolescents in need from seeking professional help include a lack of perceived need, beliefs that treatment is not effective, mistrust of providers, or stigma [32]. These concerns associated with seeking professional help probably inhibited help-seeking behavior within the ProfScreen participants as well. The variety of individual barriers cannot be fully addressed by a school-based screening and must be targeted in particular interventions. If these barriers could be successfully reduced, this might as well result in a higher effectiveness of school-based screenings regarding help-seeking rates.

Even though assignment to the ProfScreen intervention per se had no effect on follow-up at-risk status or service use, post-hoc analyses revealed that completing the ProfScreen intervention was associated with increased utilization of professional care. So, if the barriers to using the intervention can be overcome, an interview with a professional may be a good way to improve service use. However, even among the ProfScreen completers, there was no change in at-risk status. Participation per protocol in the ProfScreen intervention was therefore not able to reduce psychological stress and risk behaviour, which may have been due to the limited effect of the intervention on service use. It is also possible that the time span of one year is too short to observe effects on at-risk status. Even if the intervention improves the use of professional treatment, it may take a while for the treatment to lead to an improvement in mental health. Future studies might aim to extend the follow-up period to receive valid data regarding effects on follow-up at-risk state.

To the best of our knowledge, the ProfScreen intervention was part of the first RCT aimed to improve young people’s professional service use for mental health problems. Furthermore, it offers first findings on associations between ProfScreen completion and follow-up mental health problems and risk behaviours. However, due to the self-selection of students regarding completion of the ProfScreen intervention, we are no longer able to report results of an RCT. The screening process, including the clinical interview, was standardised and performed according to the study protocol in each country. Nevertheless, it should be noted that different countries may have different cultures, different health systems, and different barriers to care. This could lead to differences in the interview setting and the follow-up process, despite the standardization processes. However, it is likely that our findings are applicable to a wide range of European countries, and other high-income countries, with similar cultural background. Lastly, we focussed only on the students’ perspectives concerning service use. As their service use might depend on their parents, future studies could include both, the students’ and the parents’ perspectives. Further, some characteristics of the investigated subsamples must be considered when interpreting the present findings. Compared to the control group, students of the ProfScreen group screened more often positive for suicidal tendencies and problems in social relationships at baseline. A higher burden might have caused a higher treatment motivation of ProfScreen participants and could have influenced subsequent service use [23, 33]. On the other hand, students with current suicidality at baseline, experiencing the highest burden and thus a potentially increased treatment motivation, were excluded from the regular ProfScreen intervention. Although these participants were detected through the regular school-based screening, they were excluded from the usual ProfScreen procedure and received an immediate intervention, which was associated with increased follow-up service use [34]. Thus, a school-based screening might be able to increase actual help-seeking to a greater extinct than is shown by the present findings, as it identifies particularly urgent cases and can provide rapid assistance at this point.

## Conclusion

Assignment to the ProfScreen intervention as implemented within the school-based SEYLE study had no effect on professional service use nor at-risk state compared to participation in the control group. The two-stage ProfScreen intervention suffered from low participation rates in the second part, the interview for clinical evaluation by professionals. Complete participation was positively associated with follow-up service use for young people at-risk for mental problems and risk behaviours, but the intervention was only able to reach 41% of eligible students for full participation. Overall, the present study highlighted two major difficulties in school-based screenings: less than half of the sample accepted the invitation for a clinical interview, and subsequently, only few students engaged in professional treatment. Thus, prior to the implementation of large-scale school-based screening programs as a regular tool to address young people’s mental health, further evidence and improvement of interview attendance rates as well as particular interventions targeting barriers to professional help are necessary.

## Supplementary Information

Below is the link to the electronic supplementary material.

Supplementary file1 (PDF 115 KB)

Supplementary file2 (PDF 39 KB)

Supplementary file3 (PDF 94 KB)

Supplementary file4 (PDF 17 KB)

Supplementary file5 (PDF 34 KB)

## Data Availability

The data that support the findings of this study are available from the corresponding author, MK, upon reasonable request.
