# Supplementary material for: The impact of school-based screening on service use in adolescents at risk for mental health problems and risk-behaviour
Source: Eur Child Adolesc Psychiatry. 2022 Apr 30;32(9):1745–54. doi: 10.1007/s00787-022-01990-z (PMC10460322; doi:10.1007/s00787-022-01990-z)
Supplement: Supplementary file 1 — Supplementary file1 (PDF 115 KB) [file 787_2022_1990_MOESM1_ESM.pdf]

Supplement to:

The Impact of School-Based Screening on Service Use in Adolescents At-Risk for Mental Health Problems and Risk-Behaviour

European Child & Adolescent Psychiatry

Sophia Lustig, Michael Kaess*, Nina Schnyder, Chantal Michel, Romuald Brunner, Alexandra Tubiana, Jean-Pierre Kahn, Marco Sarchiapone, Christina W. Hoven, Shira Barzilay, Alan Apter, Judit Balazs, Julio Bobes, Pilar Alejandra Saiz, Doina Cozman, Padraig Cotter, Agnes Kereszteny, Tina Podlogar, Vita Postuvan, Airi Värnik, Franz Resch, Vladimir Carli, Danuta Wasserman

***Corresponding Author:** Michael Kaess, University Hospital of Child and Adolescent Psychiatry and Psychotherapy, University of Bern, Bern, Switzerland. E-Mail: Michael.Kaess@upd.ch

Online Resource 1 Screening questions and guideline-based cut-off values for at-risk students

	Instrument used/assessed items	Cut-off value	Students of ProfScreen group were invited for interview if at least one of these cut-off values was reached:
Depression	20-items of the Beck's Depression Inventory (BDI-II) [1]	Sum score according to BDI manual	BDI-score ≥ 14
Anxiety	20-item Zung Self-Rated Anxiety Scale (SAS) [2]	Sum score according to Zung manual	Zung-score ≥ 45
Suicidal tendencies	Current suicidality with a modified version of the 4-item Paykel Suicide Scale (PSS) [3]	Item 1 answer option ≥ "very often"; Items 2-4 answer option ≥ "sometimes"	Cut-off for one of the four items is reached
	Have you ever tried to take your own life (y/n)?	Lifetime suicide attempt	"Yes" is given as an answer
Non-suicidal self-injury	Lifetime non-suicidal self-harm with the 6-item version of the Deliberate Self-Harm Inventory (DSHI) [4, 5]	Answer options were coded as following: 0 "never", 1 "1-2 times", 2 "3-4 times", 3 "5 times or more"<br>The answers of questions 1-6 were summed up	sum-score ≥ 2
Eating behaviour	Body Mass Index (BMI)		BMI < 16.5
Risky behaviour (sensation seeking & delinquent behaviour)	Riding in a car with a driver that drunk alcohol (past 12 month)	1 "yes", 0 "no"	All answers were summed up, sum-score ≥ 3
	Riding skate board/roller skates in traffic or on streets without helmet (past 12 month)	1 "yes", 0 "no"	
	Riding between subway cars, holding on back of bus or other vehicles to pull you along (past 12 month)	1 "yes", 0 "no"	
	Going to places like certain streets, alleys or buildings where you know it is dangerous, such as at night or when others think something bad could happen (past 12 month)	1 "yes", 0 "no"	
	Sexual intercourse with how many people in lifetime	1 "number of people ≥ 4", 0 "number of people ≤ 3"	
	How often do you use condoms when having sexual intercourse?	1 "rarely" or "never", 0 "almost every time" or "always"	
Substance abuse	Tobacco consumption	≥2 cigarettes per day in Estonia, Germany, Hungary, Ireland, and	Reaching cut-off score for tobacco consumption

		Romania; ≥5 cigarettes per day in Italy and Slovenia; ≥7 cigarettes per day in France; and ≥10 cigarettes per day in Austria, Israel, and Spain	according to country of residence
	Alcohol consumption	(1) frequency: 2-3 times per week or more<br>(2) amount: 3-4 drinks per drinking occasion or more<br>(3) intoxication: getting 3 or more times drunk during lifetime<br>(4) hangover: having 3 or more times a hangover after drinking during lifetime	Reaching cut-off scores for (1), (2), (3), or (4)
	Illegal drug consumption	Using drugs 3 or more times during lifetime	Reaching cut-off score for drug consumption
Exposure to media	How much time do you spend during a typical day watching television, playing computer games, or surfing the internet?	Being at least 5-6 hours per day exposed to media	Reaching cut-off score for media exposure
Social relationships	How often have you felt lonely during past 12 month?	Having felt lonely “most of the time” or “always”	Reaching cut-off score for social relationships
Bullying	15 items asking about peer victimisation in the past 12 month such as “being kicked by others”, or “being teased by others”, etc. were asked. Multiple answers possible	Bullying items that were answered with yes were summed up	≥ 5 incidents
School attendance	How many times did you miss school or class during past 2 weeks without permission?	At least 3 days in the past 2 weeks.	Raching cut-off score for school attendance

Assessed items for alcohol cut-off slightly differed between baseline and follow-up. The frequency of drinking (1) and the amount (2) were not considered in the follow-up. All students that were considered at-risk for mental problems or risk-behaviour because they fulfilled cut-off for frequency of drinking (1) or amount of drinking (2) also scored on other at-risk criteria. This means that after excluding these two items from the analysis, the at-risk population consisted of the same pupils. This let us conclude that these two items are not necessary to detect at-risk states for our purpose. Missing items at follow-up concerning regularity and amount of drinking will therefore not have an impact on follow-up at-risk state that are necessary for further analyses

Additional References

1. Beck AT, Steer RA, Brown GK (1996) Beck depression inventory-II. Psychological Corporation, San Antonio, TX.

2. Zung W (1971) A rating instrument for anxiety disorders. Psychosomatics 12:371–379. https://doi.org/10.1016/S0033-3182(71)71479-0

3. Paykel, ES, Myers, JK, Lindenthal, JJ, Tanner J (1974) Suicidal feelings in the general population: a prevalence study. Brit J Psychiat, 124:460–469. https://doi.org/10.1192/bjp.124.5.460

4. Brunner R, Kaess M, Parzer P, et al (2014) Life-time prevalence and psychosocial correlates of adolescent direct self-injurious behavior: A comparative study of findings in 11 European countries. J Child Psychol Psyc, 55(4):337–348. https://doi.org/10.1111/jcpp.12166.

5. Gratz KL (2001) Measurement of deliberate self-harm: Preliminary data on the deliberate self-harm inventory. J Psychopathol Behav, 23(4), 253–263. https://doi.org/10.1023/A:1012779403943
