# Supplementary material for: The impact of school-based screening on service use in adolescents at risk for mental health problems and risk-behaviour
Source: Eur Child Adolesc Psychiatry. 2022 Apr 30;32(9):1745–54. doi: 10.1007/s00787-022-01990-z (PMC10460322; doi:10.1007/s00787-022-01990-z)
Supplement: Supplementary file 2 — Supplementary file2 (PDF 39 KB) [file 787_2022_1990_MOESM2_ESM.pdf]

Supplement to:

The Impact of School-Based Screening on Service Use in Adolescents At-Risk for Mental Health Problems and Risk-Behaviour

European Child & Adolescent Psychiatry

Sophia Lustig, Michael Kaess*, Nina Schnyder, Chantal Michel, Romuald Brunner, Alexandra Tubiana, Jean-Pierre Kahn, Marco Sarchiapone, Christina W. Hoven, Shira Barzilay, Alan Apter, Judit Balazs, Julio Bobes, Pilar Alejandra Saiz, Doina Cozman, Padraig Cotter, Agnes Kereszteny, Tina Podlogar, Vita Postuvan, Airi Värnik, Franz Resch, Vladimir Carli, Danuta Wasserman

***Corresponding Author:** Michael Kaess, University Hospital of Child and Adolescent Psychiatry and Psychotherapy, University of Bern, Bern, Switzerland. E-Mail: Michael.Kaess@upd.ch

Online Resource 2. Missing data among students that completed follow-up 2 and were not emergency cases (reduced sample see Figure 1)

Total sample (N=4,931)	Missing (n)	Missing (%)
Sex	19	0.39
Age	28	0.57
Baseline screening parameters		
Depression	48	0.97
Anxiety	139	2.82
Suicidal tendencies	150	3.04
Non-suicidal self-injury	134	2.72
Eating behaviour	373	7.56
Risky behaviour[a]	75	1.52
Substance abuse	92	1.87
Exposure to media	90	1.83
Social relationships	27	0.55
Bullying	91	1.85
School attendance	33	0.67
At-risk at baseline	431	8.74
Interview attended	0	0
Referral to further treatment	0	0
Follow-up screening parameters		
Depression	53	1.07
Anxiety	186	3.77
Suicidal tendencies	23	0.47
Non-suicidal self-injury	142	2.88
Eating behaviour	354	7.18
Risky behaviour[a]	142	2.88
Substance abuse	88	1.78
Exposure to media	261	5.29
Social relationships	41	0.83
Bullying	146	2.96
School attendance	59	1.20
At-risk at follow-up	414	8.40
Follow-up service use	0	0

[a] Sensation seeking and delinquent behaviour
