# Supplementary material for: The impact of school-based screening on service use in adolescents at risk for mental health problems and risk-behaviour
Source: Eur Child Adolesc Psychiatry. 2022 Apr 30;32(9):1745–54. doi: 10.1007/s00787-022-01990-z (PMC10460322; doi:10.1007/s00787-022-01990-z)
Supplement: Supplementary file 3 — Supplementary file3 (PDF 94 KB) [file 787_2022_1990_MOESM3_ESM.pdf]

Supplement to:

The Impact of School-Based Screening on Service Use in Adolescents At-Risk for Mental Health Problems and Risk-Behaviour

European Child & Adolescent Psychiatry

Sophia Lustig, Michael Kaess*, Nina Schnyder, Chantal Michel, Romuald Brunner, Alexandra Tubiana, Jean-Pierre Kahn, Marco Sarchiapone, Christina W. Hoven, Shira Barzilay, Alan Apter, Judit Balazs, Julio Bobes, Pilar Alejandra Saiz, Doina Cozman, Padraig Cotter, Agnes Kereszteny, Tina Podlogar, Vita Postuvan, Airi Värnik, Franz Resch, Vladimir Carli, Danuta Wasserman

*Corresponding Author: Michael Kaess, University Hospital of Child and Adolescent Psychiatry and Psychotherapy, University of Bern, Bern, Switzerland. E-Mail: Michael.Kaess@upd.ch

Online Resource 3 Type of treatment that student sought within one year (n=2,583)

	Type of treatment					
	Medication	Prof one-to-one therapy	Group therapy	Advice from health prof	Non-prof treatment[a]	No treatment
Total sample, n(%)	23 (0.9)	46 (1.8)	8 (0.3)	16 (0.6)	30 (1.16)	2,460 (95.2)
Intervention group, n(%)						
ProfScreen	10 (0.8)	29 (2.2)	5 (0.4)	9 (0.7)	17 (1.3)	1,244 (94.7)
Control	13 (1.0)	17 (1.3)	3 (0.2)	7 (0.6)	13 (1.0)	1,216 (95.8)
Referral[b], n(%) *yes*	3 (2.0)	10 (6.7)	0 (0.0)	2 (1.3)	7 (4.7)	127 (85.2)

[a] includes ‘mentor to talk to’ and ‘healthy lifestyle group’
[b] among screening completers
