# Supplementary material for: The impact of school-based screening on service use in adolescents at risk for mental health problems and risk-behaviour
Source: Eur Child Adolesc Psychiatry. 2022 Apr 30;32(9):1745–54. doi: 10.1007/s00787-022-01990-z (PMC10460322; doi:10.1007/s00787-022-01990-z)
Supplement: Supplementary file 4 — Supplementary file4 (PDF 17 KB) [file 787_2022_1990_MOESM4_ESM.pdf]

Supplement to:

The Impact of School-Based Screening on Service Use in Adolescents At-Risk for Mental Health Problems and Risk-Behaviour

European Child & Adolescent Psychiatry

Sophia Lustig, Michael Kaess*, Nina Schnyder, Chantal Michel, Romuald Brunner, Alexandra Tubiana, Jean-Pierre Kahn, Marco Sarchiapone, Christina W. Hoven, Shira Barzilay, Alan Apter, Judit Balazs, Julio Bobes, Pilar Alejandra Saiz, Doina Cozman, Padraig Cotter, Agnes Kereszteny, Tina Podlogar, Vita Postuvan, Airi Värnik, Franz Resch, Vladimir Carli, Danuta Wasserman

*__Corresponding Author:__ Michael Kaess, University Hospital of Child and Adolescent Psychiatry and Psychotherapy, University of Bern, Bern, Switzerland. E-Mail: Michael.Kaess@upd.ch

Online Resource 4 Unadjusted logistic regressions of associations between ProfScreen intervention, ProfScreen completers, age, sex, and baseline screening parameters with service use after one year

	Service use after one year		
	OR	95%-CI	N
ProfScreen group[a]	1.291	0.850-1.961	2,583
ProfScreen completer[a]	**1.761**	1.080-2.872	1,804
Age[b]	1.287	0.991-1.671	1,804
Sex[c]	1.319	0.806-2.158	1,804
Baseline screening parameters[d]			
Depression	**2.345**	1.440-3.819	1,785
Anxiety	**2.439**	1.325-4.491	1,766
Suicidal tendencies	1.618	0.949-2.759	1,782
Non-suicidal self-injury	**3.225**	1.959-5.310	1,751
Eating behaviour	0.188	0.026-1.366	1,692
Risky behaviour[e]	1.412	0.745-2.675	1,783
Substance abuse	1.010	0.672-1.801	1,776
Exposure to media	1.009	0.534-1.905	1,769
Social relationships	1.673	0.813-3.442	1,795
Bullying	**1.908**	1.070-3.405	1,772
School attendance	0.999	0.307-3.252	1,793

OR odds ratio, *CI* confidence interval, statistically significant results are displayed in **bold**

[a] Reference category: control group

[b] Reference: younger age

[c] Reference category: male

[d] Reference categories: cut-off for mental problems or risk-behaviours not met

[e] Sensation seeking and delinquent behaviour
