# Supplementary material for: The impact of school-based screening on service use in adolescents at risk for mental health problems and risk-behaviour
Source: Eur Child Adolesc Psychiatry. 2022 Apr 30;32(9):1745–54. doi: 10.1007/s00787-022-01990-z (PMC10460322; doi:10.1007/s00787-022-01990-z)
Supplement: Supplementary file 5 — Supplementary file5 (PDF 34 KB) [file 787_2022_1990_MOESM5_ESM.pdf]

Supplement to:

The Impact of School-Based Screening on Service Use in Adolescents At-Risk for Mental Health Problems and Risk-Behaviour

European Child & Adolescent Psychiatry

Sophia Lustig, Michael Kaess*, Nina Schnyder, Chantal Michel, Romuald Brunner, Alexandra Tubiana, Jean-Pierre Kahn, Marco Sarchiapone, Christina W. Hoven, Shira Barzilay, Alan Apter, Judit Balazs, Julio Bobes, Pilar Alejandra Saiz, Doina Cozman, Padraig Cotter, Agnes Kereszteny, Tina Podlogar, Vita Postuvan, Airi Värnik, Franz Resch, Vladimir Carli, Danuta Wasserman

*__Corresponding Author:__ Michael Kaess, University Hospital of Child and Adolescent Psychiatry and Psychotherapy, University of Bern, Bern, Switzerland. E-Mail: Michael.Kaess@upd.ch

Online Resource 5 Unadjusted logistic regression of association between ProfScreen intervention, ProfScreen completion, service use, age, and sex with follow-up at-risk state

	Follow-up at-risk state		
	OR	95% CI	N
ProfScreen group[a]	0.926	0.773-1.109	2,583
ProfScreen completer[a]	1.019	0.802-1.296	1,804
Service use[b]	**2.367**	1.123-4.987	1,804
Age[c]	1.115	0.987-1.259	1,804
Sex[d]	**0.736**	0.590-0.917	1,804

OR odds ratio, *CI* confidence interval, statistically significant results are displayed in **bold**
[a] Reference category: control group
[c] Reference category: no service use
[d] Reference: younger age
[e] Reference category: male
